# Lymphocyte-activation gene 3 protein expression in tumor-infiltrating lymphocytes is associated with a poor prognosis of ovarian clear cell carcinoma

**DOI:** 10.1186/s13048-023-01179-1

**Published:** 2023-05-13

**Authors:** Sumika Zaitsu, Mitsutake Yano, Sawako Adachi, Maiko Miwa, Tomomi Katoh, Yasushi Kawano, Masanori Yasuda

**Affiliations:** 1 Departments of Obstetrics and Gynecology, Oita University Faculty of Medicine, Idaigaoka 1-1, Hasama-machi, Yufu-shi, Oita, 879-5593 Japan; 2 Department of Pathology, Saitama Medical University International Medical Centre, Saitama, Japan; 3 Department of Gynecologic Oncology, Saitama Medical University International Medical Centre, Saitama, Japan

**Keywords:** Ovarian cancer, Ovarian clear cell carcinoma, Lymphocyte-activation gene 3, Immune checkpoint inhibitors, Prognosis, Tumor-infiltrative lymphocytes

## Abstract

**Background:**

Histological analysis has revealed the need for new treatment techniques for epithelial ovarian cancer. Immune checkpoint inhibitors may be a new therapeutic strategy for ovarian clear cell carcinoma (OCCC). Lymphocyte-activation gene 3 (LAG-3), an immune checkpoint, is a poor prognostic factor and a new therapeutic target for several malignancies. In this study, we demonstrated the correlation between LAG-3 expression and the clinicopathological features of OCCC. We evaluated LAG-3 expression in tumor-infiltrating lymphocytes (TILs) via immunohistochemical analysis using tissue microarrays containing surgically resected specimens from 171 patients with OCCC.

**Results:**

The number of LAG-3-positive cases was 48 (28.1%), whereas the number of LAG-3-negative cases was 123 (71.9%). LAG-3 expression significantly increased in patients with advanced stages (*P* = 0.036) and recurrence (*P* = 0.012); however, its expression did not correlate with age (*P =* 0.613), residual tumor (*P* = 0.156), or death (*P* = 0.086). Using the Kaplan − Meier method, LAG-3 expression was found to be correlated with poor overall survival (*P* = 0.020) and progression-free survival (*P* = 0.019). Multivariate analysis revealed LAG-3 expression (hazard ratio [HR] = 1.86; 95% confidence interval [CI], 1.00 − 3.44, *P* = 0.049) and residual tumor (HR = 9.71; 95% CI, 5.13 − 18.52, *P* < 0.001) as independent prognostic factors.

**Conclusion:**

Our study demonstrated that LAG-3 expression in patients with OCCC may be a useful biomarker for the prognosis of OCCC and could serve as a new therapeutic target.

## Background

Epithelial ovarian cancer is classified into several histological types based on its morphology. The pathogenesis and clinical features, including cell origin, driver genes, response to treatment, and patient prognosis, differ depending on the histological types; however, a therapeutic strategy based on the histological types has not yet been established. Ovarian clear cell carcinoma (OCCC) is resistant to existing therapies and requires a new effective therapeutic strategy. The use of immune checkpoint inhibitors (ICIs) targeting programmed cell death-1 (PD-1)/programmed death ligand-1 (PD-L1) and cytotoxic T lymphocyte-associated antigen-4 (CTLA-4) is a promising strategy for the treatment of recurrent ovarian cancer and could be more effective for OCCC than for other histological cancer types [1, 2]. Nevertheless, several limitations restrict the treatment of OCCC using ICIs, including the adverse effects of ICIs, especially the co-inhibition of CTLA-4 and PD-1 [1]. The lack of a biomarker for predicting ICI efficacy is another major problem, and data collected from experiments on the immune environment in OCCC have not provided any insights regarding this problem [3]. To overcome these limitations, we focused on a new immune checkpoint molecule.

Lymphocyte-activation gene 3 (LAG-3) is the third immune checkpoint after PD-1/PD-L1 and CTLA-4, and is expressed on the surface of several immune cells, including T cells [4]. T-cell exhaustion is characterized by the overexpression of multiple inhibitory receptors, including PD-1, CTLA-4, and LAG-3 [5]. The upregulation of LAG-3, caused by exposure to persistent inflammatory stimuli, suppresses the proliferation and activation of T cells [6, 7]. T-cell exhaustion is observed in cancers that escape from immune cells [7]. Recently, the co-inhibition of LAG-3 by relatlimab and that of PD-1 by nivolumab revealed a greater antitumor activity than that of nivolumab alone in a randomized phase III study on melanoma [8]. The results of this study also suggested that co-inhibitory LAG-3 and PD-1 are associated with fewer adverse events than co-inhibitory CTLA-4 and PD-1 [8]. Moreover, co-inhibition of LAG-3 and PD-1 enhanced antitumor activity compared with single-ICI in a mouse model of solid tumors, such as melanoma, colon adenocarcinoma, and fibrosarcoma [7]. Co-inhibition of LAG-3 and PD-1 is more effective and safer than traditional ICI therapy.

Matsuzaki et al. [9] observed that tumor-infiltrating lymphocytes (TILs) expressing LAG-3 in ovarian cancer decreased the effector function of PD-1^+^ CD8^+^ T cells and restored the effector function under co-inhibition of LAG3 and PD-1 in vitro. However, no studies have demonstrated the effect of LAG-3 expression on the prognosis of patients with OCCC. To the best of our knowledge, this study is the first to determine the relationship between LAG-3-positive TILs and their clinical features as well as the potential of LAG-3 as a new therapeutic target and clinical biomarker of OCCC.

## Results

### Patients’ characteristics

The patients’ characteristics are depicted in Table 1. The age of the 171 patients ranged from 32 to 80 years; the mean age was 55.3 years. Of the 171 patients, 79 (46.2%) were aged < 55 years and 92 (56.1%) were aged ≥ 55 years. The classification of patients according to the International Federation of Gynecology and Obstetrics (FIGO) staging was variable: 111 patients were in stage I (64.9%), 25 in stage II (14.6%), 31 in stage III (18.1%), and 4 in stage IV (2.3%). A total of 146 patients (85.4%) underwent complete resection, whereas 25 (14.6%) underwent incomplete resection. Adjuvant chemotherapy was administered to 129 (75.4%) patients. The regimens are as follows: paclitaxel and carboplatin were administered to 77 patients, docetaxel and carboplatin to 35, irinotecan and cisplatin to 12, and gemcitabine and carboplatin to 1 patient. Forty-four patients (25.7%) experienced recurrence, whereas 127 (74.3%) did not experience it. Thirty-two patients (18.7%) died, and 139 (81.3%) survived.

**Table 1:** Characteristics of patients with OCCC (*N* = 171)

Characteristic	*N* (%)
**Age (years)**	
Median (range)	55.3 (32 − 80)
<55	79 (46.2)
≥55	92 (54.1)
**FIGO stage**	
I	111 (64.9)
II	25 (14.6)
III	31 (18.1)
IV	4 (2.3)
**Surgical procedures**	
TAH + BSO + OM	142 (83.0)
BSO + OM	6 (3.5)
USO + OM	8 (4.7)
OM	15 (8.8)
**Surgical status**	
Complete resection	146 (85.4)
Incomplete resection	25 (14.6)
**Adjuvant chemotherapy**	
Yes	129 (75.4)
Paclitaxel + Carboplatin	77
Docetaxel + Carboplatin	35
Irinotecan + Cisplatin	12
Gemcitabine + Carboplatin	1
Not available	4
No	42 (24.6)
**Recurrence**	
Yes	44 (25.7)
No	127 (74.3)
**Death**	
Yes	32 (18.7)
No	139 (81.3)

Abbreviations: OCCC, ovarian cell carcinoma; FIGO, International Federation of Obstetrics and Gynecology; TAH, total abdominal hysterectomy; BSO, bilateral salpingo-oophorectomy; OM, omentectomy; USO, unilateral salpingo-oophorectomy

### Correlation between clinicopathological features and LAG-3 expression

Table 2 presents the correlation between patient characteristics and LAG-3 expression. There were 48 LAG-3-positive cases (28.1%) and 123 (71.9%) LAG-3-negative cases. LAG-3 expression significantly increased in patients with advanced stages, FIGO stages III and IV (*P* = 0.036), and in cases of cancer recurrence (*P* = 0.012). However, no correlation was observed between LAG-3 expression and age (*P* = 0.613), residual tumor (*P* = 0.156), and death (*P* = 0.086).

**Table 2:** Correlations between patients’ characteristics and LAG-3 expression assessed via immunohistochemistry

		LAG-3		
Characteristic		Positive	Negative	*P*
ALL		48	123	
Age, years (median = 55.3)	< 55	67	56	0.613
	≥ 55	24	24	
FIGO stage	I + II	33	103	0.036*
	III + IV	15	20	
Surgical residual tumor	Yes	10	15	0.156
	No	38	108	
Adjuvant chemotherapy	Yes	37	92	0.845
	No	11	31	
Recurrence	Yes	19	25	0.012*
	No	29	98	
Death	Yes	13	19	0.086
	No	35	104	

*statistical significance; *P*-value < 0.05

Abbreviations: LAG-3, lymphocyte activation gene-3 protein; FIGO, International Federation of Obstetrics and Gynecology

### Correlation between patients’ survival and LAG-3 expression

**Fig. 1:**
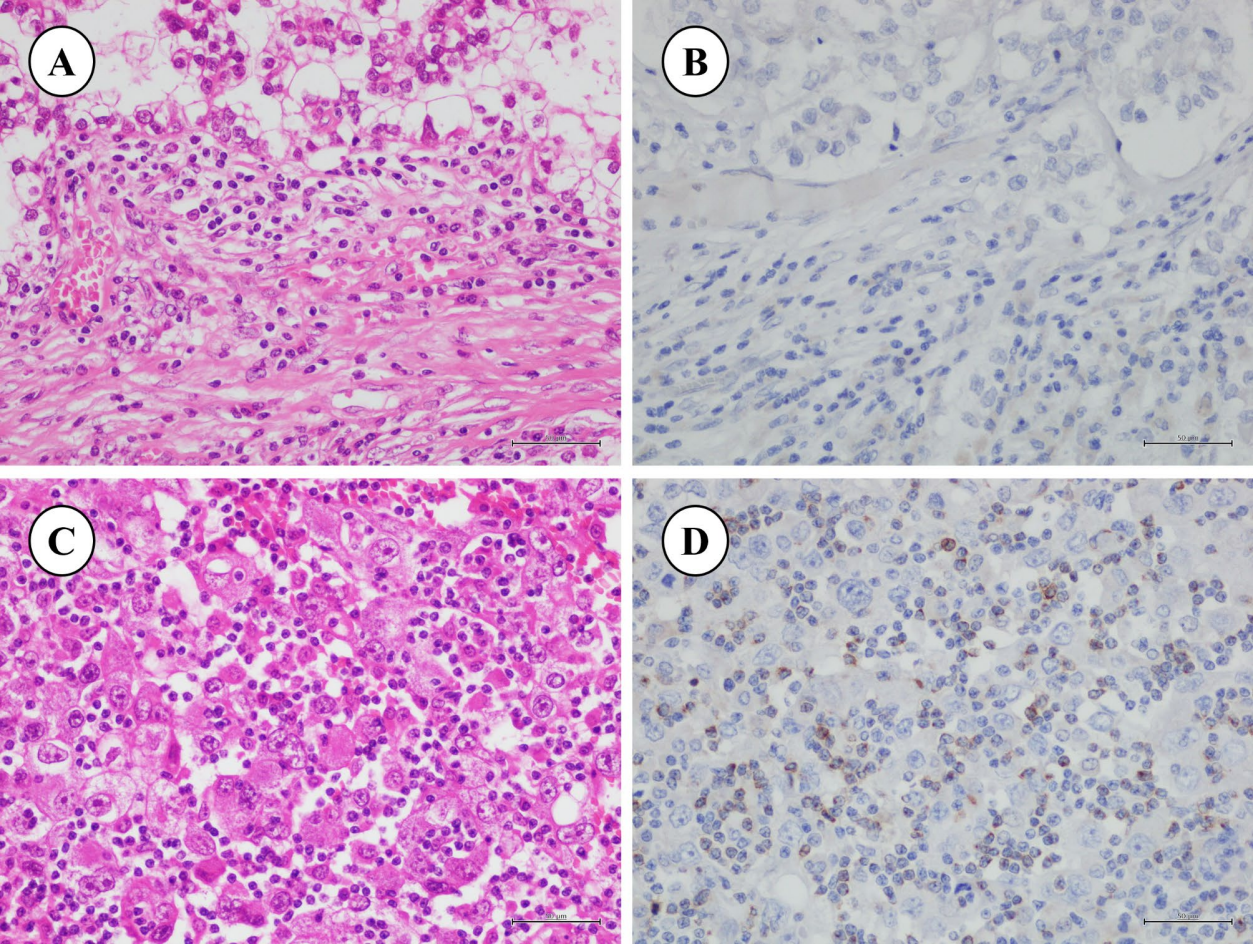
Immunohistochemical expression of the lymphocyte activation gene-3 protein (LAG-3) in ovarian clear cell carcinoma (OCCC). (**a**, **b**) LAG-3-negative case: LAG-3 expression was observed in < 20% of the total tumor-infiltrative lymphocytes. (a: H & E, ×40; b: LAG-3, ×40); (**c**, **d**) LAG-3-positive case: LAG-3 expression was observed in > 20% of total tumor-infiltrative lymphocytes. (a: H & E, ×40; b: LAG-3, ×40). OCCC cells did not express LAG-3.

Kaplan − Meier curve analysis (Fig. 1) revealed that poorer overall survival (OS) (*P* = 0.020) and progression-free survival (PFS) (*P* = 0.019) were correlated more with LAG-3 expression than with no LAG-3 expression. The univariate and multivariate analyses of PFS with Cox regression analysis were also used to evaluate the correlation (Table 3). The univariate analysis indicated that advanced FIGO stages (stage III and IV; hazard ratio [HR] = 5.37; 95% confidence interval [CI], 2.95 − 9.77, *P* < 0.001), LAG-3 expression (HR = 2.53; 95% CI, 1.39 − 4.61, *P* = 0.002), and residual tumor (HR = 11.11; 95% CI, 5.95 − 20.83, *P* < 0.001) were prognostic factors for PFS. The multivariate analysis regarded LAG-3 expression (HR = 1.86; 95% CI, 1.00 − 3.44, *P* = 0.049) and residual tumor (HR = 9.71; 95% CI, 5.13 to 18.52, *P* < 0.001) as independent prognostic factors.

**Table 3:** Univariate and multivariate analyses for PFS in patients with OCCC.

	Univariate analysis				Multivariate analysis		
Characteristic	HR	95% CI	*P*		HR	95% CI	*P*
Age (years)	0.88	0.49 − 1.60	0.679				
FIGO stage	5.37	2.95 − 9.77	< 0.001*				
LAG-3	2.53	1.39 − 4.61	0.002*		1.86	1.00‒3.44	0.049*
Residual tumor	11.11	5.95 − 20.83	< 0.001*		9.71	5.13‒18.52	< 0.001*

*statistical significance; *P*-value < 0.05

Abbreviations: PFS, progression-free survival; OCCC, ovarian clear cell carcinoma; HR, hazard ratio; CI, confidence interval; FIGO, International Federation of Obstetrics and Gynecology; LAG-3, lymphocyte activation gene-3 protein

## Discussion

To the best of our knowledge, this study was the first to investigate the correlation between LAG-3 expression and poor prognosis in OCCC. We demonstrated that LAG-3 expression was observed in higher FIGO stages and was related to poor OS and PFS. LAG-3 expression is notably independent of prognostic factors for PFS. Patients with OCCC for a longer period presented higher expression of LAG-3 than those with OCCC for a shorter period. Conversely, several studies have demonstrated that LAG-3 expression is correlated with a good prognosis [10–14]. This discrepancy can be explained by the immunomodulatory as well as immune exhaustive functions of LAG-3. Soluble LAG-3 separated from the cell surface has been reported to activate dendritic cells [15]. In contrast, the upregulation of LAG-3 is a poor prognostic factor in most malignancies [16–25]. This result was consistent with the function of LAG-3 in exhausting T cells. Khalique et al. [3] suggested that regulatory T cells were excluded from the vicinity of tumor cells in low-risk patients, indicating that high-risk patients have a more immunosuppressive microenvironment than that in low-risk patients. LAG-3-expressing regulatory T cells exhibited an enhanced immune suppressive function, supporting the hypothesis that the presence of LAG-3 on TILs in patients with cancer leads to poor survival [10]. These results reflect that LAG-3 negatively regulates immune cells secondary to long-term exposure to inflammation [6, 7]. The results of previous studies on various cancers focusing on the relationship between LAG-3 expression analyzed using immunohistochemistry and/or immunofluorescence and patient prognosis are summarized in Table 4 [10–14, 16–35]. These results and review in this study revealed that LAG-3 on TILs is a biomarker for poor prognosis and a potential therapeutic target in advanced/recurrent OCCC.

**Table 4:** Correlation between LAG-3 expression and prognosis in various malignancies

Article	Type of cancer	Classification	Case	Method	Prognosis
Zaitsu S. (present study)	Ovarian cancer	Clear cell carcinoma	171	IHC	poor OS and PFS
Victor S. (2021)	Breast cancer	Variety (after neoadjuvant therapy)	66	IHC	No significant difference
Maximilian J. M. (2021)	Glioma	Variety	97	IHC	No significant difference
Artem G. (2021)	Classic Hodgkin lymphoma	Hodgkin lymphoma	15	IHC	No significant difference
Chubin L. (2021)	Liver cancer	Sarcomatoid HCC	31	IHC	No significant difference
Kristiina K. (2021)	Classic Hodgkin lymphoma	Hodgkin lymphoma	131	IHC	No significant difference
Lena S. (2021)	Pancreatic cancer	Pancreatic ductal carcinoma	69	multiplex IF	poor DFS
John A. (2021)	Osteosarcoma	Pulmonary metastasis	25	IHC	poor PFS
Luo F. (2021)	Nasopharyngeal carcinoma	Variety	182	IHC	poor DFS
Gaëlle Rhyner Agocs (2021)	Colon cancer	Stage II	142	IHC	favorable DFS
Takeuchi M. (2020)	Adult T cell leukemia/lymphoma	Variety	69	IHC	No significant difference
Mengzhou G. (2020)	Liver cancer	HCC	143	IF	poor OS and DFS
Gebauer F. (2020)	Esophageal cancer	Adenocarcinoma	421	IHC, IF	favorable OS
Xiao S. (2020)	Thyroid cancer	Medullary thyroid cancer	200	IHC	No significant difference
Félix B. (2020)	Ovarian cancer	Variety	98	IHC	No significant difference
Ila D. (2019)	Lung cancer	Variety (after ICI treatment via PD-1)	90	multiplexed QIF	poor PFS
Christoph M. (2019)	Nasopharyngeal carcinoma	Variety	30	IHC	No significant difference
Wenjia W. (2019)	Esophageal cancer	SCC	261	IHC	poor OS and RFS
Hong W. (2019)	Oral carcinoma	SCC (before nimotuzumab therapy)	36	IHC	poor OS
Fucikova J. (2019)	Ovarian cancer	HGSC	80	IHC	favorable RFS and OS
Jingjing D. (2018)	Esophageal	SCC (after esophagectomy)	55	IHC	No significant difference
Sigurd M. (2017)	Lung cancer	Variety	500	IHC	favorable DSS
Burugu S. (2017)	Breast cancer	Variety	3992	IHC	favorable CSS
Yayi H. (2017)	Lung cancer	Variety	139	IHC	poor OS and PFS
Wei-Wei D. (2016)	Head and neck cancer	SCC	194	IHC, IF	poor OS (negative LN metastasis)
Giulia B. (2016)	Breast cancer	Triple-negative (after adjuvant chemotherapy)	104	IHC	poor OS and RFS

LAG-3, lymphocyte activation gene 3; HCC, hepatocyte carcinoma; ICI, immune checkpoint therapy; PD-1, programmed cell death 1; SCC, squamous cell carcinoma; HGSC, high-grade serous cell carcinoma; IHC, immunohistochemistry; IF, immunofluorescence; QIF, quantitative immunofluorescence; OS, overall survival; PFS, progression-free survival; DFS, disease-free survival; DFS, disease-free survival; DSS, disease-specific survival; CSS, cancer-specific survival; LN, lymph node

LAG-3 has an inhibitory pathway, which differs from that of other immune checkpoints, such as PD-1/PD-L1 and CTLA-4. Co-inhibition of PD-1 and CTLA-4 enhances not only antitumor activity but also autoimmune diseases [36], as observed in mice with PD-1 or CTLA-4 deficiencies [37, 38]. Unlike PD-1 and CTLA-4, LAG-3 single deficiency was not a factor of autoimmune disease without an autoimmune background and had less antitumor activity. LAG-3 induces autoimmune diseases and enhances antitumor activity by co-inhibiting PD-1 [9, 39]. Co-inhibition of PD-1 and LAG-3 may cause fewer adverse events than co-inhibition of PD-1 and CTLA-4, which is characterized by single-deficient autoimmune disease. Co-inhibition of PD-1 and CTLA-4 has an antitumor effect in patients with OCCC, but with severe adverse events [1, 2]; however, a randomized phase III study on melanoma revealed that co-inhibition of PD-1 and LAG-3 leads to favorable outcomes [8]. These results indicate that a more suitable target for co-inhibition with PD-1 is LAG-3 rather than CTLA-4. As OCCC with LAG-3 on TILs is advanced and has a poor prognosis, LAG-3 is a promising therapeutic target. Furthermore, ICIs are effective against platinum-resistant cells [1]. Therefore, ICIs via LAG-3 may spread to platinum-resistant ovarian cancer.

This study has two limitations. First, owing to the use of tissue microarray, we were unable to examine the whole tissue, although we analyzed a large number of patients. Second, we analyzed LAG-3, but not its ligands. LAG-3 is currently being developed as a novel therapeutic target; however, it is not clear which ligands should be targeted for cancer therapy. In the future, research on the ligands that contribute to downregulation of immune cells in cancers is warranted.

## Conclusions

In summary, LAG-3 expression in patients with OCCC is a useful biomarker for predicting prognosis. This study also revealed that LAG-3 can be a potential therapeutic target and an effective biomarker for predicting therapeutic efficacy in OCCC.

## Materials and methods

### Patients and samples

A total of 171 patients diagnosed with OCCC underwent surgery at Saitama Medical University International Medical Centre between 2007 and 2016. All study participants provided informed consent (or a formal waiver of consent). Patient data, including age, recurrence/PFS, death/OS, FIGO stage, surgical status (complete resection or incomplete resection), and treatment methods, were reviewed. All samples were collected without neoadjuvant chemotherapy. This study was approved by the Institutional Review Board of the Ethics Committee of the Saitama Medical University International Medical Centre, (IRB number, 16–257) and was performed in accordance with the guidelines of the Helsinki Declaration of 1975, as revised in 1983.

### Immunohistochemical staining

The immunohistochemical expression of LAG-3 was analyzed using a tissue microarray (KIN-2, AZUMAYA, Tokyo, Japan). Tissue microarrays consisted of paraffin-embedded tissue blocks constructed by extracting 3-mm cylindrical tissue cores, with appropriate histological findings. All blocks were sliced into Sect. (4 μm thick). After deparaffinization and heat-induced antigen retrieval using 1 mM ethylenediaminetetraacetic acid (pH 9.0), all sections were incubated with 3% hydrogen peroxidase for 10 min to quench the endogenous peroxidase activity. These sections were further incubated with DAKO Blocking Reagent (Protein Block Serum-Free Ready-to-use [Code X0909], DAKO North America Inc., California, USA) at room temperature for 20 min to block nonspecific antigens. LAG-3 antibody (Ab180187, clone EPR4392, diluted 1:1,500; Abcam) was applied as the primary antibody at 4 °C overnight. Secondary antibodies (HISTOFINE Simple Stain MAX-PO MULTI [NICHIREI Code 424,151], Nichirei Biosciences Inc., Tokyo, Japan) were applied at room temperature for 45 min. The antigen/antibody complex formation was performed in 3,3ʹ-diaminobenzidine tetrahydrochloride and hydrogen peroxidase substrate solution for 10 min and counterstained with hematoxylin for 5 s.

### Interpretation of immunohistochemical results

**Fig. 2:**
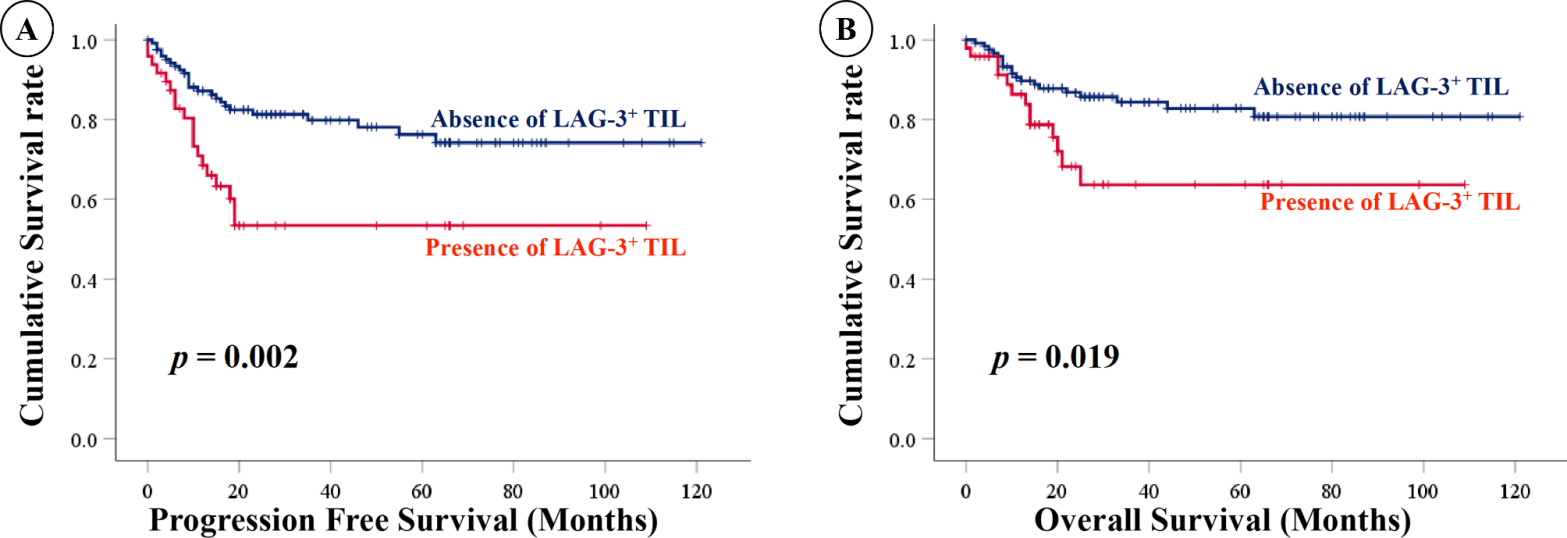
Correlation between the expression of lymphocyte-activation gene 3 (LAG-3) and overall survival (OS)/progression-free survival (PFS). The correlation was obtained using Kaplan − Meier statistical analysis and log-rank test in patients with ovarian clear cell carcinoma (OCCC). (**a**) PFS and LAG-3 expression; (**b**) OS and LAG-3 expression

Immunohistochemical staining was performed by two researchers (SZ and MiY) who were blinded to the patients’ data. We defined brown staining of > 20% of TILs as positive LAG-3 expression (Fig. 2) [25].

### Statistical analysis

Statistical analysis was performed using SPSS version 25.0 (SPSS Inc., Chicago, IL, USA). We assessed the correlation between the expression of LAG-3 and patient data using Pearson’s chi-square test or Fisher’s exact test. The Kaplan − Meier method was used to estimate survival curves. The log-rank test was used to assess the differences between the groups. The Cox proportional hazards model was used to analyze univariate and multivariate survival. Statistical significance was set at *P* < 0.05.

## Data Availability

The datasets used and/or analyzed during the current study are available from the corresponding author on reasonable request.

## Abbreviations

CI: confidence interval
CTLA-4: cytotoxic T lymphocyte-associated antigen-4
FIGO: International Federation of Gynecology and Obstetrics
HR: hazard ratio
ICI: immune checkpoint inhibitor
LAG-3: lymphocyte activation gene-3
OS: overall survival
OCCC: ovarian clear cell carcinoma
PFS: progression-free survival
PD-1: programmed cell death-1
PD-L1: programmed death ligand-1
TIL: tumor-infiltrating lymphocyte
